# Impact of Vehicle Composition on Solubility, Autoclave Sterilization Stability, and Antibacterial Activity of Ciprofloxacin Hydrochloride Ear Drops

**DOI:** 10.3390/ijms26094458

**Published:** 2025-05-07

**Authors:** Weerasak Samee, Sarin Tadtong, Wanna Eiamart, Pattaraporn Singwiset, Sirivimon Yingyomsarn, Patcharawee Nunthanavanit

**Affiliations:** 1 Department of Pharmaceutical Chemistry, Faculty of Pharmacy, Srinakharinwirot University, Nakhon Nayok 26120, Thailand; pattaraporn.siws@gmail.com (P.S.); sirivimon.yingyomsarn@gmail.com (S.Y.); patcharw@g.swu.ac.th (P.N.); 2 Department of Pharmacognosy, Faculty of Pharmacy, Srinakharinwirot University, Nakhon Nayok 26120, Thailand; sarin@g.swu.ac.th; 3 Chula Pharmacokinetic Research Center, Faculty of Medicine, Chulalongkorn University, Bangkok 10330, Thailand; wanna.e@chula.ac.th

**Keywords:** ciprofloxacin, solubility, stability, ear drops, sterilization, chromatography, antibacterial

## Abstract

This research focused on the formulation of ciprofloxacin hydrochloride ear drops for the treatment of acute otitis externa, caused by pathogens such as *Staphylococcus aureus* and *Pseudomonas aeruginosa*. The study aimed to enhance the solubility of ciprofloxacin at physiological pH and improve its stability during autoclave sterilization by employing polyalcohol vehicle systems composed of deionized water, glycerin, and propylene glycol. Formulations containing 0.33% *w*/*v* ciprofloxacin HCl were evaluated for solubility within a pH range of 4.0 to 7.0 and underwent autoclave sterilization to assess their chemical stability and antibacterial efficacy. Following sterilization, the formulations were stored at 25 °C in amber containers for a duration of 180 days. High-performance liquid chromatography (HPLC) was utilized to evaluate chemical stability, while antibacterial activity was determined using the disk diffusion method. The results demonstrated that glycerin and propylene glycol effectively inhibited ciprofloxacin precipitation at physiological pH. The ciprofloxacin content decreased by less than 3.58% while preserving antimicrobial efficacy against *S. aureus* and *P. aeruginosa*. Both formulations retained over 90% of their labeled drug content, indicating a minimum shelf life of 180 days under the specified storage conditions.

## 1. Introduction

Bacterial infections of the ear, particularly chronic suppurative otitis media (CSOM), pose significant global health challenges, leading to serious complications like hearing loss. The etiology of these infections is predominantly bacterial, with pathogens such as *Staphylococcus aureus* and *Pseudomonas aeruginosa* frequently identified in studies from regions including Tanzania, Nigeria, and India [1,2,3]. This highlights the necessity for effective antimicrobial treatments to manage infections and prevent complications. Ciprofloxacin, a fluoroquinolone antibiotic, has become a valuable option for treating ear infections, especially in the form of ear drops. Research has demonstrated that ciprofloxacin ear drops effectively target the primary bacteria associated with CSOM, particularly *Pseudomonas aeruginosa*, which often exhibits antibiotic resistance [2,4]. While ciprofloxacin can be considered a first-line treatment for select populations, the emergence of bacterial resistance remains a critical concern [5,6,7]. Research indicates that earwax contains fatty acids, cholesterol, and antimicrobial peptides that inhibit pathogen growth [8,9]. Earwax has demonstrated both antibacterial and antifungal properties, crucial for preventing infections within the external auditory canal [9,10,11]. Glycerin and propylene glycol are commonly used cerumenolytic vehicles. Glycerin acts as a humectant, attracting moisture to soften cerumen, thereby facilitating its removal [12]. Similarly, propylene glycol serves as a solvent that enhances the penetration of cerumenolytic agents into impacted earwax [13]. Both agents are recognized for their safety and efficacy, making them suitable for both adults and children [14,15]. It is generally accepted that using a combination of cerumenolytic agents improves the likelihood of complete wax clearance compared to untreated cases. Glycerin and propylene glycol, when combined, enhance earwax removal by effectively softening and dissolving the impacted cerumen [10,16].

The stability of ciprofloxacin is influenced by several factors, including temperature, pH, and the presence of excipients or metal ions. Research has indicated that ciprofloxacin remains stable under refrigeration and at body temperature; however, at room temperature, its stability is limited, particularly in mixed solutions, highlighting the importance of storage conditions [17]. Prajaputra et al. [18] found that ciprofloxacin remained stable at temperatures ranging from 11 °C to 30 °C over a six-day period, suggesting that this temperature range is suitable for clinical handling. Hubicka et al. [19] demonstrated that metal ions in acidic solutions can cause degradation, influenced by both the type of metal ion and temperature. This necessitates careful formulation design to preserve stability. Furthermore, formulation strategies can enhance ciprofloxacin stability [20]. Photostability is another vital factor, as Al-Mardini and Mando [21] found that ciprofloxacin is susceptible to degradation under light exposure in tablet and eye drop formulations. Therefore, protecting ciprofloxacin formulations from light is essential to maintaining their efficacy.

Fluoroquinolone antibiotics are currently being formulated as otic solutions; however, these medications are prohibitively expensive in Thailand due to the absence of domestic production, as they are neither manufactured locally nor included in the national essential drug list. This situation results in significant costs when prescribed. Consequently, hospitals often rely on a 0.33% *w*/*v* ciprofloxacin HCl (equivalent to 0.3% *w*/*v* ciprofloxacin) injection administered as ear drops. This approach presents several challenges, notably the formation of a white precipitate of ciprofloxacin, which arises from its zwitterionic properties that promote precipitation within a pH range of approximately ± 2 of its two pKa values (the strongest acidic pKa1 value of 5.56 and the strongest basic pKa2 value of 8.77) [22]. The pH of a healthy ear canal typically ranges between 5.0 and 5.7, which can contribute to this precipitation issue. Furthermore, the efficacy of the injectable formulation is compromised by its water-soluble nature, limiting its ability to penetrate and effectively target pathogens embedded in cerumen, a waxy substance that accumulates in the ear.

This study aims to develop a 0.33% *w*/*v* ciprofloxacin HCl ear drops formulation by transitioning the vehicle from a glycerin-borax solution to one primarily composed of glycerin or propylene glycol. This strategy is designed to minimize ciprofloxacin precipitation across a range of physiological pH levels found in the ear. Preparing the ear drops formulation under aseptic conditions is crucial to ensure both safety and efficacy. Due to the high viscosity of the formulation, filtration through a 0.2-micron membrane presents challenges. Thus, moist heat sterilization via autoclaving is considered a viable alternative, although the heat applied may cause ciprofloxacin degradation. Consequently, this research investigates the effects of the formulation vehicle and moist heat sterilization on the chemical stability and antimicrobial efficacy of the ciprofloxacin HCl ear drops against *S. aureus* and *P. aeruginosa*.

## 2. Results

### 2.1. Effect of pH on Solubility of Ciprofloxacin

**Figure 1:**
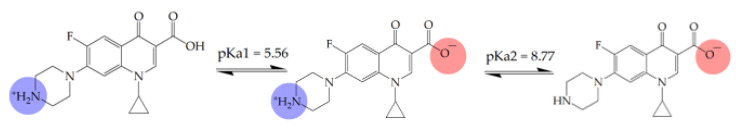
Anionic form of the carboxylic group (pKa1 = 5.56), cationic form of the secondary amine group (pKa2 = 8.77) and switter ionic form in the structure of ciprofloxacin.

According to the DRUGBANK database [22], ciprofloxacin exhibits a pKa1 of 5.56, indicating its strongest acidic property, and a pKa2 of 8.77, reflecting its strongest basic characteristic. The compound has a water solubility of 1.35 g/L. Theoretically, ciprofloxacin is likely to precipitate in its zwitterionic form within a pH range of approximately 3.56 to 10.77, calculated as pH = pKa ± 2, depending on various concentrations (as illustrated in Figure 1). Additionally, the water-soluble form of ciprofloxacin is ciprofloxacin HCl, which exists in a cationic state at a pH of approximately 4.0. This zwitterionic form of ciprofloxacin has the potential to precipitate at the pH of the ear canal, which ranges from 5.0 to 5.7.

Understanding how pH affects ciprofloxacin precipitation is essential for optimizing its solubility in pharmaceutical formulations and minimizing the risk of precipitation, which can influence bioavailability. The relationship between pH and the precipitation of ciprofloxacin was determined utilizing the principles of acid-base chemistry and solubility product calculations. For a 0.33% ciprofloxacin HCl (equivalent to 0.3% ciprofloxacin) solution, the precipitation pH (pHp) can be calculated as follows:

From DRUGBANK: Molecular weight of ciprofloxacin = 331.34 g/mol; Solubility of ciprofloxacin = 1.35 g/L; pKa1 = 5.56

Using the modification equation of a weak acid for calculated switter ion precipitation:

pHp = pKa + log[S_0_]/[S – S_0_]

The molar solubility (S_0_) of ciprofloxacin was calculated as:

S_0_ = 1.35 g/L × 1 mole/331.34 = 4.0744 × 10^−3^ moles/L or M

The concentration (S) for a 0.3% solution was calculated as:

S = 0.3 g/100 mL × 1000 mL/L × 1 mole/331.34 g = 9.0541 × 10^−3^ moles/L or M

Substituting the values, we have:

pHp = 5.56 + log 0.0040774/(0.00905 − 0.00407) = 5.47

Thus, it can be concluded that a 0.3% ciprofloxacin solution will precipitate at or above a pH of 5.47.

**Figure 2:**
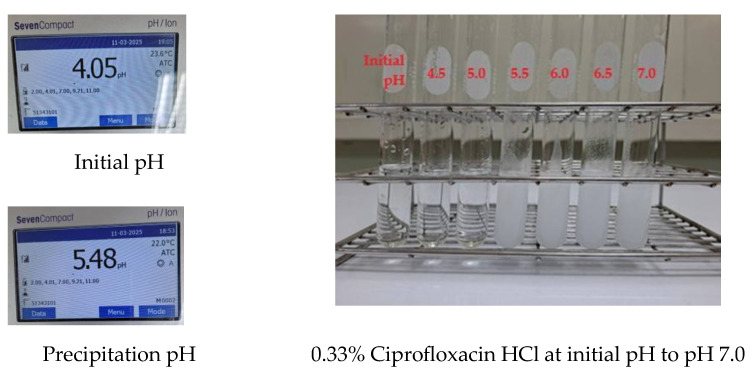
Physical appearance of 0.33% ciprofloxacin HCl from initial pH to pH 7.0.

To confirm the solubility of ciprofloxacin under physiological pH conditions, a 0.33% ciprofloxacin HCl solution was prepared using deionized water as the solvent (designated as Vehicle A). The pH was measured and recorded at 4.03 ± 0.03. To evaluate the solubility of this formulation across a pH gradient up to pH 7.0, titrations were conducted using a 0.1 N sodium hydroxide (NaOH) solution. As shown in Figure 2, the 0.33% ciprofloxacin HCl exhibited precipitation at a pH of 5.48, which closely aligns with the calculated precipitation pH (pHp) of 5.47. Importantly, the resulting suspension remained stable even as the pH increased to 7.0. Consequently, these results indicate the necessity for alternative formulations to enhance ciprofloxacin solubility at physiological pH.

### 2.2. Vehicle Selection for Improveing Solubility of Ciprofloxacin at Physiological pH

The Hansen solubility parameters (HSP) serve as an essential tool for solvent selection, systematically describing the interactions between solvents and solutes by segmenting total solubility into three distinct parameters: dispersion (δ_d_), polarity (δ_p_), and hydrogen bonding (δ_h_) [23,24]. As shown in Table 1, the calculated δ_d_, δ_p_ and δ_h_ of ciprofloxacin was 25.6695, 12.8938 and 15.7950, respectively.

The primary outcome of the HSP framework was the distance (R_a_) between the Hansen spheres of a solute and solvent, which quantified their interactions and miscibility. This distance was a critical determinant of the compatibility between various solvents and solutes. Lower R_a_ values indicated higher miscibility and stronger molecular interactions between the selected solvents [23]. In Table 2, the R_a_ values for ciprofloxacin in relation to water, glycerin, and propylene glycol are 33.55, 19.58, and 21.68, respectively. These results suggest that ciprofloxacin demonstrated greater solubility in glycerin and propylene glycol compared to water, which could be attributed to the lower R_a_ values associated with these solvents. Most commercial formulations include propylene glycol in concentrations ranging from 5% to 50%, with a typical concentration between 5% and 20%. Given concerns regarding the ototoxicity associated with propylene glycol [25], a concentration of 20% was chosen for this study. A mixture comprising 10% purified water and 90% glycerin was designated as Vehicle B, whereas a combination of 10% purified water, 70% glycerin, and 20% propylene glycol was designated as Vehicle C. As presented in Table 2, the calculated R_a_ values for Vehicles B and C were 22.76 and 22.14, respectively.

**Figure 3:**
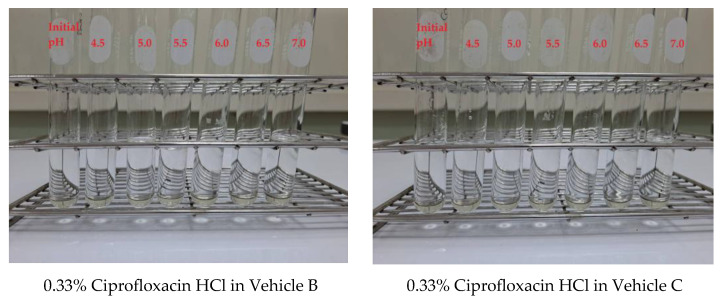
Physical appearance of 0.33% ciprofloxacin HCl using Vehicle B and Vehicle C from initial pH to pH 7.0.

Due to the high viscosity of glycerin, a 3.3% ciprofloxacin HCl aqueous solution was initially prepared and subsequently incorporated into glycerin or a mixture of glycerin and propylene glycol to create 0.33% ciprofloxacin HCl solutions. The pH of the 0.33% ciprofloxacin HCl solution in both Vehicle B and Vehicle C was measured at 4.03 ± 0.03, which was comparable to the solution prepared using Vehicle A. As illustrated in Figure 3, the 0.33% ciprofloxacin HCl formulations utilizing Vehicle B and Vehicle C remained clear within the physiological pH range of 4.0 to 7.0 which were satisfied to be the 0.33% ciprofloxacin HCl ear drops.

These results indicate that 90% glycerin and the combination of 70% glycerin with 20% propylene glycol effectively prevent the precipitation of 0.33% ciprofloxacin hydrochloride formulations at neutral pH. This finding confirms that HSP serves as effective tools for selecting suitable vehicles for ciprofloxacin ear drops formulations.

### 2.3. Validation of Analytical Method for Determination of Ciprofloxacin Hydrochloride Eardrops

**Figure 4:**
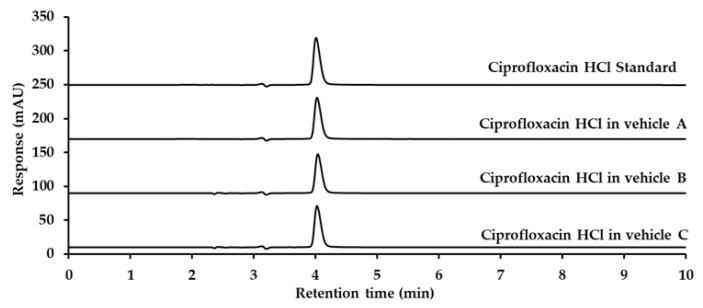
Chromatograms of ciprofloxacin HCl standard, ciprofloxacin hydrochloride in Vehicle A, Vehicle B, and Vehicle C, detected at 278 nm using UV detection.

The analytical method for ciprofloxacin analysis was modified in accordance with the United States Pharmacopoeia 45—National Formulary 40 (USP 45-NF 40) [26], as detailed in Section 4.5. The retention time for ciprofloxacin was approximately 4.0 min, with no interference from eardrop vehicles, as demonstrated in Figure 4.

**Figure 5:**
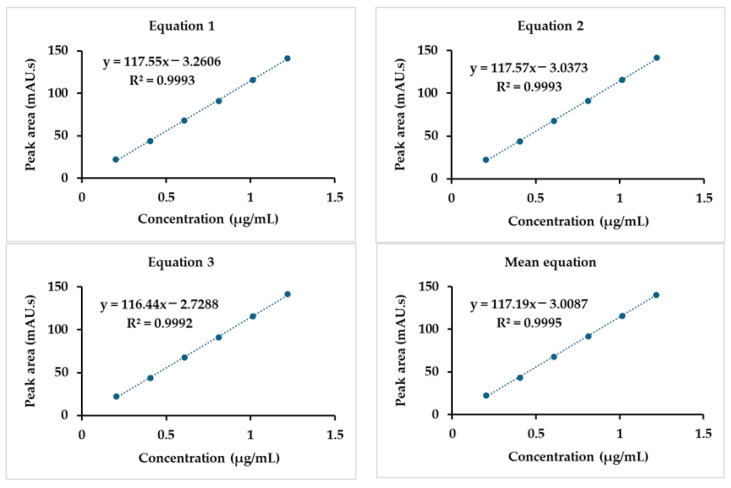
Calibration curves for ciprofloxacin HCl.

The developed analytical method underwent system suitability testing and method validation to ensure compliance with the standards established in the ICH Harmonisation for better health [27] and the AOAC Guideline 2016 [28]. The analysis of the spiked ciprofloxacin HCl standard solution in a mixture of Vehicle B and Vehicle C at a concentration of 0.6 µg/mL was conducted six times under the specified HPLC analysis conditions. The system suitability for ciprofloxacin HCl was found to be within the acceptance limit, as the percentage standard deviation (% RSD) of retention time and peak area was less than 2%. The number of theoretical plates is a direct indicator of the efficiency of a chromatographic column. An increased number of theoretical plates signifies a more efficient separation process, leading to sharper and more resolved peaks during the elution of analytes. In this study, the number of theoretical plates was determined to be 13,265, which exceeded the analytical method requirement of 2500. Tailing factors are used to quantify the symmetry of chromatographic peaks, thereby indicating the extent of analyte resolution. The tailing factor for ciprofloxacin was measured at ±1.13, which is below the required threshold of ±1.5. The resolution was not found due to the single peak of ciprofloxacin HCl in the chromatogram. The results indicated that the method met the acceptance criteria set forth in the ICH guidelines, as presented in Table 3. The method validation results were as follows:

Linearity: The peak areas of the ciprofloxacin HCl were plotted against the corresponding concentration. As presented in Figure 5, the calibration curves for ciprofloxacin HCl were found to be linear in the concentration range of 0.2–1.2 µg/mL with the correlation coefficient of 0.9995, which meet to requirement of >0.995.

Limit of Detection (LOD) and Limit of Quantitation (LOQ): Based on the HPLC analysis, the LOD and LOQ were calculated by multiplying three times and ten times the standard deviation of the y-intercept and divided by the mean slope of the linear equation. Considering the values from all three linear equations, the standard deviation of the y-intercept was determined to be 0.218, while the average slope of the linear equations was 117.19. Consequently, the LOD and LOQ concentrations were established at 6.14 ng/mL and 18.61 ng/mL, respectively.

Accuracy: The mean % recovery of the spiked ciprofloxacin HCl standard solution in a mixture of Vehicle B and Vehicle C at concentrations of 0.3, 0.6, and 0.9 µg/mL was 99.60%, 100.83% and 100.22%, respectively. All values were within the acceptable limit of 95–105% as specified by the AOAC Guideline 2016.

Precision: The precision of the analytical method was evaluated through the analysis of the ciprofloxacin HCl standard solution at concentrations of 0.3, 0.6, and 0.9 µg/mL. The results indicate that the %RSD for intra-day repeatability ranged from 0.12% to 1.19%, while the %RSD for inter-day intermediate precision was 1.04%. Both values were within the acceptable limit of less than 2% as specified by the AOAC Guideline 2016.

The method validation results indicated that the method met the acceptance criteria set forth in the AOAC Guideline 2016, as presented in Table 4.

### 2.4. Stability of 0.33% Ciprofloxacin HCl Ear Drops Following Autoclave Sterilization

In moist heat sterilization, a method extensively utilized in hospitals settings, specific combinations of pressure and temperature are essential for the effective inactivation of microorganisms. In this study, the conditions for pressurized steam sterilization were set at 15 min at 121 °C and 1 kg/cm^2^ [29]. Five amber glass bottles containing 0.33% ciprofloxacin HCl ear drops formulations were sterilized using autoclaving. The chemical stability of the formulations was evaluated through HPLC, and their antimicrobial efficacy was assessed against *S. aureus* and *P. aeruginosa* using the agar diffusion assay.

#### 2.4.1. Chemical Stability of 0.33% Ciprofloxacin HCl Ear Drops Under Sterilization Conditions

**Figure 6:**
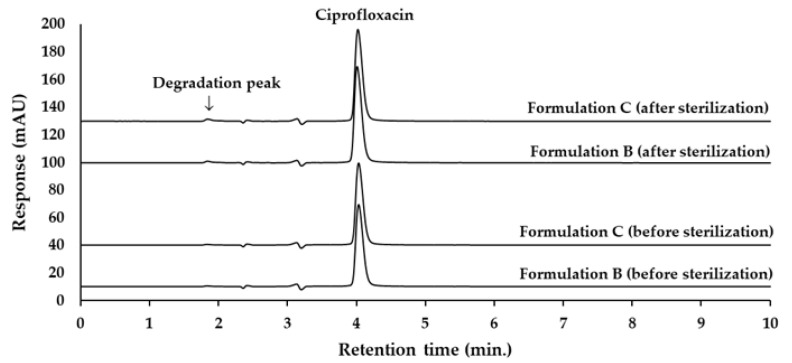
Chromatograms of 0.33% ciprofloxacin HCl ear drops formulation B and formulation C, before and after sterilization, detected at 278 nm using UV detection.

The HPLC analysis results of ciprofloxacin concentration in ear drops formulations B and C were compared before and after the sterilization process. The calculated % labeled amount before sterilization for formulation B and formulation C was 103.12 ± 0.83% and 104.73 ± 1.15%, respectively. After sterilization, the HPLC chromatograms of both formulations revealed a small degradation peak at a retention time of 1.9 min (Figure 6).

The degradation peak eluted with the solvent front, which was expected to contain the polar molecules present in that region. Long et al. (2023) [30] suggested pathways for the degradation products of ciprofloxacin via photocatalytic degradation. Among these, the smallest identified degradation product was 2-butylamine. In the presence of 0.1% formic acid within the mobile phase, 2-butylamine was protonated, resulting in the formation of positively charged ammonium ions. This cationic small molecule did not retain on the C18 column, facilitating its easy elution with the acidic mobile phase. Consequently, the degradation product observed in the chromatogram was likely to be 2-butylamine.

**Figure 7:**
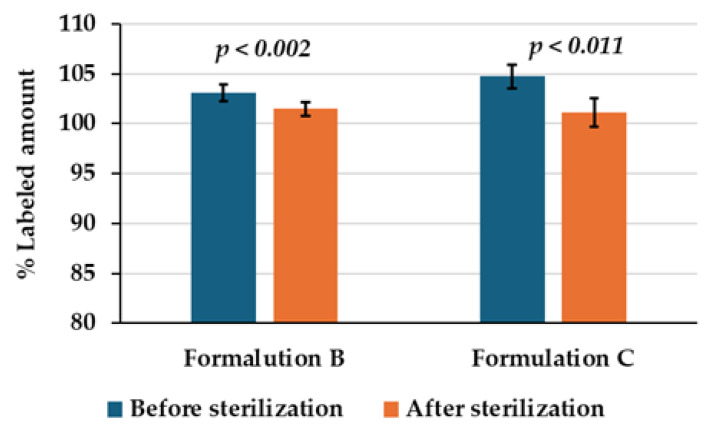
Stability of 0.33% ciprofloxacin HCl ear drops formulations B and C under autoclave sterilization.

The % labeled amount for formulation B and formulation C was measured at 101.50 ± 0.68% and 101.15 ± 1.41%, respectively. Formulation B and formulation C exhibited decreases in % labeled amount of 1.62% and 3.58%, respectively, as presented in Table 5 and Figure 7. Statistical analysis was conducted using IBM SPSS Statistics version 26, employing a paired samples *t*-test at a 95% confidence interval. The analysis indicated a statistically significant difference (*p* < 0.05) in the % labeled amount of ciprofloxacin before and after sterilization for both formulations. This finding suggested that steam sterilization at 121 °C for 15 min may have resulted in the degradation of ciprofloxacin. However, it is noteworthy that both formulations maintained an acceptable % labeled amount range of 90–110% post-sterilization, in accordance with the standards set by USP 45 NF 40.

#### 2.4.2. Antimicrobial Efficacy of 0.33% Ciprofloxacin HCl Ear Drops

**Figure 8:**
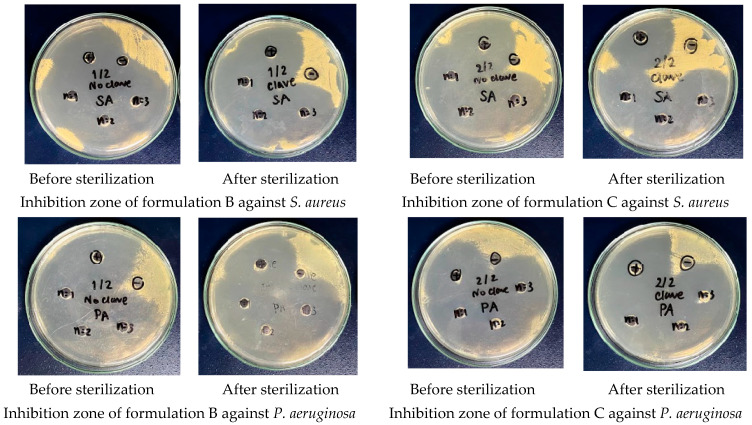
Inhibition zone against *S. aureus* and *P. aeruginosa* for 0.33% ciprofloxacin HCl formulations B and C, before and after sterilization.

The antimicrobial efficacy of ear drops formulations B and C were evaluated by comparing their capacity to inhibit microbial growth before and after the sterilization process. This assessment employed the agar diffusion method, which involved measuring the diameter of the inhibition zone. The results revealed that the diameter of the inhibition zone against *S. aureus* and *P. aeruginosa* for both formulations was 40.00 ± 0.00 mm (Figure 8). No significant differences were observed between the groups prior to and following sterilization. Consequently, it can be concluded that steam sterilization at 121 °C for 15 min did not adversely affect the antimicrobial efficacy of both ear drops formulations.

### 2.5. Storage Stability of 0.33% Ciprofloxacin HCl Ear Drops

**Figure 9:**
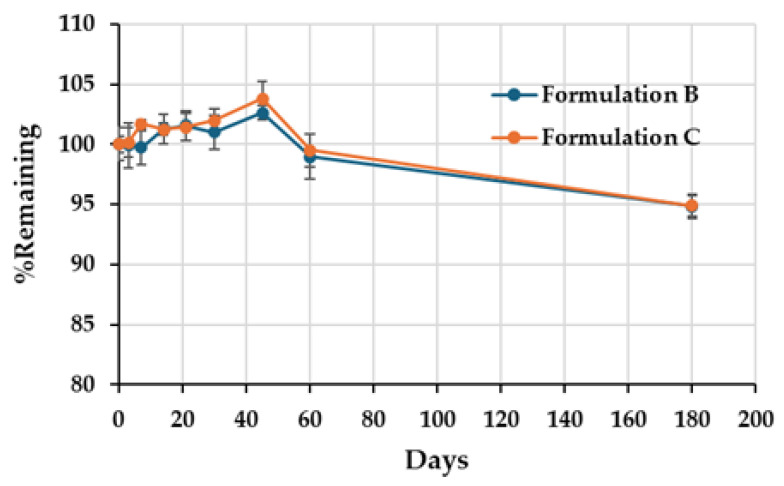
Stability of 0.33% ciprofloxacin HCl formulations B and C under storage conditions.

The recommended shelf life of commercial ciprofloxacin ear drops is 24 months when unopened (without the dropper assembly in place) and 14 days once opened with the dropper assembly attached. Special storage precautions include maintaining the product below 25 °C, protecting it from light, and avoiding refrigeration of both the unopened and opened bottles. Clark MP et al. reported that ciprofloxacin retained its efficacy against both *S. aureus* and *P. aeruginosa* for at least four months after opening [31]. In the present study, both formulations of 0.33% ciprofloxacin HCl ear drops were stored in amber glass bottles to shield them from light and maintained at 25 °C. The ciprofloxacin content was analyzed at intervals of 0, 3, 7, 21, 30, 45, 60, and 180 days post-sterilization. The percentage of labeled amount remained above 90% at day 180, in accordance with the standards established by USP 45 NF 40 (Table 6 and Table 7 and Figure 9). The percentage of drug remaining was calculated as follows:

$$\%\;Remaining=\frac{\%\;labeled\;amount\;on\;a\;later\;day}{\%\;labeled\;amount\;on\;Day\;0}\times 100$$

Statistical analysis was performed using Microsoft Excel 365, utilizing a one-way ANOVA analysis with a 95% confidence interval. The results revealed a statistically significant difference (*p* < 0.05) in storage days for both formulations.

**Figure 10:**
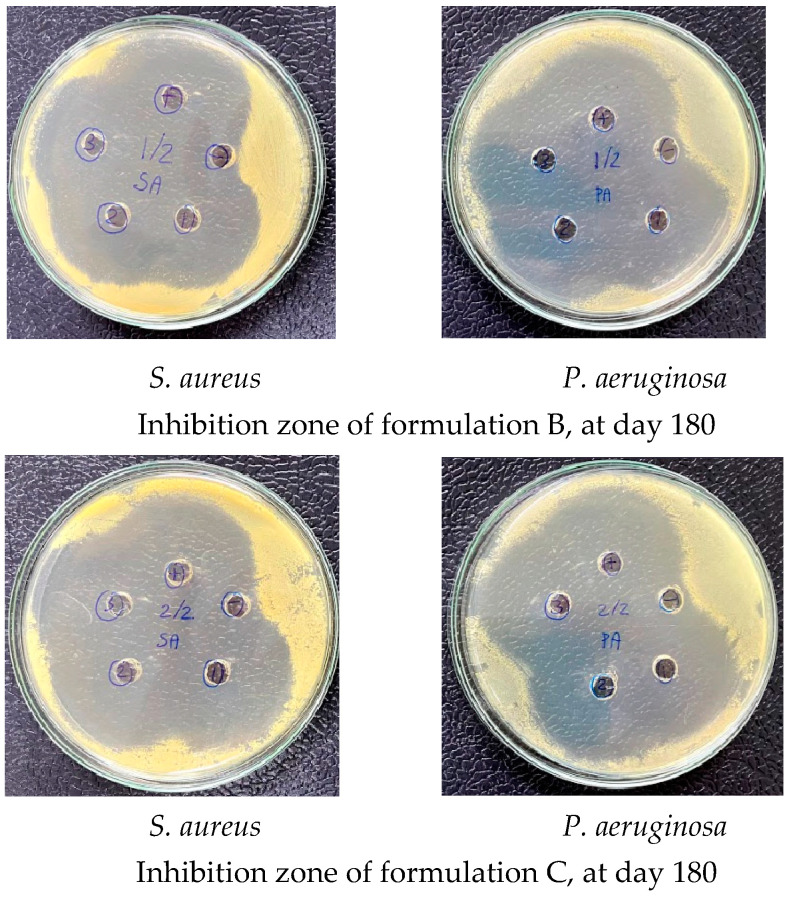
Inhibition zone against *S. aureus* and *P. aeruginosa* for 0.33% ciprofloxacin HCl formulations B and C at day 180.

On day 180, the inhibition zones against *S. aureus* and *P. aeruginosa* for both formulations were measured at 40.00 ± 0.00 mm (Figure 10). These findings indicate that the 0.33% ciprofloxacin HCl ear drops in both formulations preserved their chemical stability and antimicrobial efficacy for up to 180 days following preparation.

## 3. Discussion

This study aimed to create a 0.33% *w*/*v* ciprofloxacin HCl ear drops formulation by modifying the vehicle from a glycerin-borax solution to one that predominantly consisted of glycerin or propylene glycol. The rationale behind this modification was to reduce ciprofloxacin precipitation across various pH levels. Glycerin is known for its high hygroscopicity, enhancing moisture retention, which is crucial for maintaining the stability of the formulation [32,33]. As glycerin concentration increases, its water-retention capacity improves, aiding in the prevention of ciprofloxacin precipitation under varying pH conditions. Additionally, propylene glycol is similarly hygroscopic and can maintain the solubility of drugs in formulations, supporting the goal of minimizing precipitation [32]. The findings of this study demonstrate that both glycerin and propylene glycol effectively inhibit the precipitation of ciprofloxacin within the physiological pH range of 4.0 to 7.0, which corresponds to the conditions of the middle ear.

Maintaining aseptic conditions during the formulation process is paramount for ensuring both the safety and efficacy of ear drops. Given the high viscosity of the formulation, traditional sterilization methods, including the use of a 0.2-micron filter, may present challenges. Thus, moist heat sterilization via autoclaving is proposed as an alternative method. However, this technique can potentially lead to the degradation of ciprofloxacin due to the applied heat, necessitating further investigation into the effects of moist heat on the chemical stability and antimicrobial efficacy of the formulation [34,35]. Various studies indicate that sterilization parameters, such as temperature and duration, are critical for determining the effectiveness of moist heat sterilization without compromising drug integrity [34,35,36]. This study found that the moist heat sterilization at 121 °C for 15 min may have resulted in the degradation of ciprofloxacin, which decreased the %labeled amount of 3.58%.

Furthermore, the antimicrobial efficacy of the ciprofloxacin formulation against common pathogens such as *S. aureus* and *P. aeruginosa* is of significant interest. Ciprofloxacin, a fluoroquinolone antibiotic, exhibits broad-spectrum activity against these microorganisms. Its efficacy can be influenced by both the formulation vehicle and the sterilization process, necessitating a thorough evaluation of the resulting formulation’s stability and antimicrobial performance [35,37]. Previous research suggests that the choice of vehicle and sterilization method can significantly affect a drug’s bioavailability and its efficacy against bacterial strains, a concern that this study aims to address [34,35]. This study confirmed that the 0.33% ciprofloxacin HCl formulations maintained their antimicrobial against *S. aureus* and *P. aeruginosa* after sterilization and during extended storage conditions over a period of 180 days.

The formulation of ciprofloxacin ear drops using liposomes has garnered significant attention due to its potential to enhance drug delivery while mitigating the adverse effects associated with conventional dosage forms. Liposomal formulations can improve drug solubility, extend the duration of action, and facilitate targeted delivery, making them particularly advantageous in the treatment of conditions such as chronic suppurative otitis media. The incorporation of liposomes into ciprofloxacin ear drops aligns with the increasing emphasis on optimizing antibiotic therapy for chronic conditions. Kiakojuri et al. reported enhanced therapeutic outcomes and improved hearing thresholds in patients treated with ciprofloxacin drops, underscoring the practical implications of employing such formulations in clinical settings [38,39]. The preparation techniques are critical to the overall success of liposomal formulations. A key aspect of the formulation process is the characterization of liposomes, which includes the evaluation of their size, stability, and drug encapsulation efficiency. Techniques such as cryo-transmission electron microscopy (cryo-TEM) have been utilized to visualize the distribution of ciprofloxacin nanocrystals within liposomes, confirming the solid-state crystallization of the drug [40,41]. Liposomal formulations have been demonstrated to enhance the solubility of ciprofloxacin. However, the complexities associated with their preparation processes and quality control can present significant challenges in hospital environments. Consequently, the formulation of a ciprofloxacin solution may serve as a more economical and practical alternative for the extemporaneous preparation of ciprofloxacin ear drops in these settings.

Given the concerns about the ototoxic side effects of propylene glycol, we recommend a formulation consisting of 90% glycerin, which is safer and more convenient to prepare in a hospital setting. The preparation process for 100 mL of 0.33% ciprofloxacin HCl ear drops involves dissolving 0.33 g of ciprofloxacin HCl in 10 mL of water, followed by the addition of 90 mL of glycerin, and stirring the mixture until a homogeneous solution is achieved. The solution is then transferred into an amber bottle, sealed with a plastic cap, and autoclaved at 121 °C for 15 min. Following sterilization, the containers should be stored in dark areas at temperatures between 15 and 30 °C. Under these conditions, the unopened containers maintained chemical stability and antimicrobial efficacy over a period of 180 days.

## 4. Materials and Methods

### 4.1. Materials

Ciprofloxacin HCl standard and formic acid were purchased from Sigma-Aldrich^®^ Co. (St. Louis, MO, USA), ciprofloxacin HCl raw material was obtained from Anhui Medipharm Co., Ltd. (Hefei, China). Acetonitrile of chromatography grade was purchased from Merck (Darmstadt, Germany). Glycerin and propylene glycol were acquired from Chemiphan corporation Co., Ltd. (Bankok, Thailand). Microbials *S. aureus* DMST8013 and *P. aeruginosa* DMST15501 obtained from Department of Medical Sciences, Ministry of Public Health (Nonthaburi, Thailand).

### 4.2. Calculation of Hansen Solubility Parameters

The Hansen Solubility Parameters (HSPs) for ciprofloxacin were calculated using the group contribution method based on its molecular structure. The values for dispersion force (δ_d_), dipolar intermolecular force (δ_p_), and hydrogen bonding energy (δ_h_) were obtained using Equations (1), (2), and (3), respectively:

(1) $$\delta_{d}=\left(\sum_{i}N_{i}C_{i}+W\sum_{j}M_{j}D_{j}+17.3231\right)\;{MPa}^{1/2}$$

(2) $$\delta_{p}=\left(\sum_{i}N_{i}C_{i}+W\sum_{j}M_{j}D_{j}+7.3548\right)\;{MPa}^{1/2}$$

(3) $$\delta_{h}=\left(\sum_{i}N_{i}C_{i}+W\sum_{j}M_{j}D_{j}+7.9793\right)\;{MPa}^{1/2}$$

In these equations, C_i_ represents the contribution of the first-order group of type i, which appears N_i_ times in the target structure, while D_j_ denotes the contribution of the second-order group of type j, appearing M_j_ times. The constant W is set to 0 for compounds lacking second-order groups and to 1 for those that contain them.

Solvents with HSP values that closely align with those of ciprofloxacin were chosen for vehicles. The compatibility between a solvent and the solute of interest can be assessed using the radius (R_a_), calculated as per Equation (4).

(4) $$R_{a}^{2}=4{\left(\delta_{di}-\delta_{dj}\right)}^{2}+{\left(\delta_{pi}-\delta_{pj}\right)}^{2}+{\left(\delta_{hi}-\delta_{hj}\right)}^{2}$$

where i refers to the HSPs of the solute, and j refers to the HSPs of the solvent.

### 4.3. Preparation of 0.33% Ciprofloxacin HCl Eardrops

First, 0.33 g of ciprofloxacin HCl (equivalent to 0.3 g of ciprofloxacin) was weighed and subsequently transferred into a 250 mL beaker. Ten milliliters of distilled water was added, and the mixture was stirred until fully dissolved. Three types of vehicles were incorporated: Vehicle A, which consisted of purified water; Vehicle B, which contained 90 mL of glycerin; and Vehicle C, which was a mixture of 70 mL of glycerin and 20 mL of propylene glycol. The mixture was stirred until uniform. Each formulation of the 0.33% *w*/*v* ciprofloxacin HCl ear drops was then dispensed into 50 mL amber bottles, with each bottle labeled according to the corresponding formulation number. The prepared formulations were subjected to sterilization using moist heat at 121 °C for 15 min.

### 4.4. Determination of the Effect of pH on Ciprofloxacin Solubility

In this experiment, 100 milliliters of a prepared 0.33% *w*/*v* ciprofloxacin HCl solution was subjected to a titration process. The solution was stirred continuously using a magnetic stirrer to ensure uniform mixing during the titration. A 0.1 N sodium hydroxide solution was gradually added to the ciprofloxacin solution. The addition of sodium hydroxide served to raise the pH of the solution incrementally. During the titration, the pH was monitored continuously using a calibrated pH meter, which provided real-time readings of the pH level as the alkali was added. Throughout the procedure, the appearance of the solution was carefully observed for any signs of precipitation. Precipitation may occur when the solubility limit of ciprofloxacin is exceeded as the pH changes, potentially forming insoluble ciprofloxacin zwitterion. When precipitation was observed, the corresponding pH value was recorded. The data collected, including the pH values at which precipitation occurred, were analyzed to determine how changes in pH affected the solubility of ciprofloxacin.

### 4.5. HPLC Analysis of Ciprofloxacin Ear Drops

According to the United States Pharmacopoeia 45—National Formulary 40 (USP 45-NF 40) [26], the method for analyzing the quantity of ciprofloxacin hydrochloride via HPLC utilizes a stationary phase of an L1 column, with dimensions of 4.6 mm × 25 cm, and a mobile phase comprising acetonitrile and 0.025 M phosphoric acid in a 13:87 ratio, at a flow rate of 1.5 mL/min. The column temperature is maintained at 30 ± 1 °C, the injection volume is set at 10 µL, and detection occurs at a wavelength of 278 nm. The HPLC system employed in this study was an Agilent 1260 Infinity II series (Santa Clara, CA, USA). This system included a quaternary pump, an autosampler, a multi-column thermostat, and a photodiode array detector. Chromatographic separation was performed using an ACE 5 C18-AR column (4.6 × 250 mm, 5 µm) obtained from Aberdeen, Scotland, in conjunction with a Phenomenex C18 guard column (4 mm × 3 mm × 5 µm) from Torrance, CA, USA. The mobile phase consisted of 0.1% formic acid in acetonitrile (designated as phase A) and 0.1% formic acid in purified water (designated as phase B). Samples were pretreated and analyzed using isocratic elution composed of 23% phase A and 77% phase B over a duration of 10 min. The analysis was carried out at a flow rate of 1 mL/min, with a detection wavelength range of 200–600 nm, specifically including a wavelength of 278 nm. The column temperature was maintained at 25 °C, and the injection volume was set to 20 µL.

### 4.6. Method Validation

The developed HPLC method was validated based on the AOAC guidelines, assessing parameters such as linearity, limit of detection (LOD), limit of quantification (LOQ), accuracy, and precision.

#### 4.6.1. Specificity

The specificity of the HPLC analysis was assessed by comparing the results from the ciprofloxacin HCl standard solution with the corresponding sample solutions at a specific wavelength of 278 nm. It was confirmed that the standard peak was distinctly resolved from other peaks in the chromatograms of the samples, as evidenced by their respective retention times. Further validation of the specificity for both the standard and sample solutions was conducted through triplicate analyses.

#### 4.6.2. Linearity

Six-point calibration curves were constructed using specified concentrations of ciprofloxacin HCl ranging from 0.2 to 1.2 µg/mL, with a correlation coefficient observed to be over 0.995, in compliance with AOAC guidelines. All measurements were performed in triplicate.

#### 4.6.3. Limit of Detection (LOD) and Limit of Quantification (LOQ)

Using the data from the linear equations, the limit of detection (LOD) and limit of quantification (LOQ) were determined with the following formulas:

LOD = 3.3 × (Standard Deviation of Y-intercept)/(Mean of Slope)

LOQ = 10 × (Standard Deviation of Y-intercept)/(Mean of Slope)

The LOD and LOQ values were calculated as 3.3 and 10 times the ratio of the standard deviation of the calibration curve to the mean slope of the calibration curve, respectively.

#### 4.6.4. Accuracy

The recovery of the developed HPLC method was assessed using spiked samples at three different concentration levels of ciprofloxacin: 0.3, 0.6, and 0.9 μg/mL. Recovery percentages were determined by comparing the observed concentrations with the known concentrations of the spiked samples. The accuracy of the method was validated through triplicate analyses.

#### 4.6.5. Precision

The precision of the method was assessed through repeatability by carrying out triplicate injections consecutively within a single day at low, medium, and high concentration levels. To evaluate intermediate precision, six injections were performed over three different days at a medium concentration level. The percentage relative standard deviation (%RSD) of the mean recovery percentage (% recovery) was calculated to determine the method’s precision.

### 4.7. Evaluation of Antimicrobial Activity of 0.33% Ciprofloxacin HCl Ear Drops

#### 4.7.1. Preparation of Microbial Cultures

Bacterial strains *S. aureus* DMST8013 and *P. aeruginosa* DMST15501 were cultured in tryptic soy broth (TSB) at 37 °C for 18 h. The suspension of the bacteria was then prepared in sterile water to achieve a turbidity equivalent to McFarland No. 5.

#### 4.7.2. Antimicrobial Well Diffusion Assay

A 100 µL suspension of the prepared microbial culture was introduced into pre-poured tryptic soy agar plates with wells created using a 6 mm diameter cylinder cup. A sterile cotton swab was subsequently employed to distribute the inoculum evenly across the surface of the agar. The test substance, consisting of a 0.33% *w*/*v* ciprofloxacin HCl ear drops formulation, was added to the wells, ensuring they were completely filled. Sterile water served as the negative control, while a positive control consisting of 3.3 mg/mL ciprofloxacin HCl was also added to the wells at a volume of 100 µL. The test plates were incubated at 37 °C for 18 h, after which the diameters of the inhibition zones were measured. This experiment was carried out over three independent trials, with one replicate per trial (n = 3, 1 replicate). Observations and results were recorded using a Vernier caliper to measure the inhibition zones. The average inhibition zone was then calculated and reported as the mean ± SD.

### 4.8. Statistical Analysis

Data are expressed as mean, SD, and RSD (%), using Microsoft Excel 365 software (Microsoft Co., Redmond, WA, USA). Statistical analysis was conducted using IBM SPSS Statistics version 26 (IBM, Armonk, NY, USA).

## 5. Conclusions

This research examined modifications in the vehicle composition of ciprofloxacin ear drops to address the precipitation issue of ciprofloxacin at the pH level of the ear canal while maintaining sterility through moist heat sterilization. These adjustments were deemed essential for ensuring drug stability and antimicrobial activity, which are critical for patient safety and treatment outcomes. The findings demonstrated that both glycerin and propylene glycol effectively prevented the precipitation of ciprofloxacin within a physiological pH range of 4.0 to 7.0, which is relevant for the conditions in the ear canal. Furthermore, the results indicated that moist heat sterilization at 121 °C for 15 min may result in the degradation of ciprofloxacin, leading to a reduction in the labeled amount by 3.58%. Nevertheless, the study confirmed that the 0.33% ciprofloxacin HCl formulations retained their antimicrobial efficacy against *S. aureus* and *P. aeruginosa* after sterilization and throughout an extended storage period of 180 days.

## Figures and Tables

**Table 1:** Prediction of Hansen Solubility Parameters of ciprofloxacin.

Group	N_i_	C_i_			(N_i_C_i_)p	(N_i_C_i_)d	(N_i_C_i_)h
1st-Order		**δ**_d_	**δ**_p_	**δ**_h_			
COOH	1	−0.291	0.9042	3.7390	−0.2910	0.9042	3.7390
ACF	1	0.1170	0.1856	−0.7182	0.1170	0.1856	−0.7182
ACH	3	0.1105	−0.5303	−0.4305	0.3315	−1.5909	−1.2915
>C=O	1	−0.4343	0.7905	1.8147	−0.4343	0.7905	1.8147
-CH2N	2	0.8116	0.9412	1.3400	1.6232	1.8824	2.6800
>N [1] (in cyclic)	3	0.2218	−2.2018	−0.0452	0.6654	−6.6054	−0.1356
-CH2NH	2	1.4681	2.8345	1.2505	2.9362	5.6690	2.5010
AC	4	0.8446	0.6187	0.0084	3.3784	2.4748	0.0336
Ring of 3 carbons	1	0.0200	1.8288	−0.8073	0.0200	1.8288	−0.8073
ΣN_i_C_i_					8.3464	5.5390	7.8157
Constant (C)					17.3231	7.3548	7.9793
ΣN_i_C_i_ + C					25.6695	12.8938	15.7950

**Table 2:** Calculated radius (R_a_) of HSP values of ciprofloxacin with solvents.

Name	Hansen Solubility Parameters			R_a_
Solute	**δ**_d_	**δ**_p_	**δ**_h_	
Ciprofloxacin	25.6695	12.8938	15.7950	
Solvent				
Water (Vehicle A)	15.5	16	42.3	33.55
Propylene glycol	16.8	9.4	23.3	19.58
Glycerin	17.2	12	29.3	21.68
Vehicle B	17.03	12.4	30.6	22.76
Vehicle C	16.95	11.88	29.4	22.14

**Table 3:** System suitability results of the analytical method.

Parameters	% RSD of Retention Time	% RSD of Peak Area	Resolution	Number of Theoretical Plate	Tailing Factor
Acceptance criteria	≤2%	≤2%	>6	2500	±1.5
Results	0.06	0.11	**−**	13,265	±1.13

**Table 4:** Method validation results of the analytical method.

Linearity	Linear Equation			R^2^	LOD (ng/mL)	LOQ (ng/mL)
1	Y = 117.55X − 3.0626			0.9993	6.14	18.61
2	Y = 117.57X − 3.0373			0.9993		
3	Y = 116.44X − 2.7288			0.9992		
mean	Y = 117.19X − 3.0087			0.9995		
Day	Concentration (µg/mL)		%Recovery	Mean recovery	SD	%RSD
	Actual	Found				
1	0.3105	0.3126	100.68	99.73	0.8341	0.84
	0.3025	0.2998	99.11			
	0.3005	0.2987	99.40			
	0.6210	0.6206	99.94	100.22	1.1958	1.19
	0.6050	0.6001	99.19			
	0.6010	0.6102	101.53			
	0.9315	0.9235	99.14	99.24	0.1166	0.12
	0.9075	0.9003	99.21			
	0.9015	0.8958	99.37			
2	0.6025	0.6134	101.81	99.68	1.6571	1.66
	0.6015	0.6087	101.20			
	0.6005	0.6002	99.95			
	0.6000	0.5947	99.12			
	0.6015	0.5854	97.32			
	0.6100	0.6021	98.70			
3	0.6075	0.6025	99.18	99.72	0.6344	0.64
	0.6050	0.6003	99.22			
	0.6025	0.5986	99.35			
	0.6075	0.6104	100.48			
	0.6100	0.6135	100.57			
	0.6050	0.6022	99.54			
Inter-Day				99.71	1.0361	1.04

**Table 5:** Stability of 0.33% ciprofloxacin HCl eardrops under sterilization conditions.

Sample	%Labeled Amount			
	Before Sterilization		After Sterilization	
	Formulation B	Formulation C	Formulation B	Formulation C
1	104.31	105.06	102.27	102.71
2	103.37	105.44	101.73	99.19
3	102.98	104.41	100.87	102.23
4	102.91	102.91	101.93	100.42
5	102.01	105.84	100.70	101.19
Mean ± SD	103.12 ± 0.83	104.73 ± 1.15	101.50 ± 0.68 *	101.15 ± 1.41 *

* Significant differences between before and after sterilization (*p* < 0.05).

**Table 6:** Percentage of labeled amount and %remaining of ciprofloxacin HCl formulation B.

Sample	%Labeled Amount								
	Day 0	Day 3	Day 7	Day 14	Day 21	Day 30	Day 45	Day 60	Day 180
1	102.27	100.55	100.68	101.45	101.27	102.96	105.79	99.77	95.12
2	101.73	100.82	101.01	101.80	103.77	102.79	105.57	99.49	95.88
3	100.87	102.47	101.50	102.59	103.20	102.19	102.78	99.87	96.05
4	101.93	102.95	101.54	104.09	102.84	103.56	103.59	100.24	97.46
5	100.70	100.17	101.33	104.10	104.28	101.02	102.96	102.88	96.71
Mean *	101.50	101.39	101.21	102.81	103.07	102.50	104.14	100.45	96.24
SD	0.6816	1.24	0.36	1.25	1.15	0.96	1.44	1.38	0.92
	%Remaining								
Mean *	100	99.89	99.72	101.29	101.55	100.99	102.60	98.97	94.82
SD	0.67	1.22	0.36	1.23	1.13	0.95	1.42	1.36	0.87

* Significant differences between days (*p* < 0.05) were determined using a one-way ANOVA.

**Table 7:** Percentage of labeled amount and %remaining of ciprofloxacin HCl formulation C.

Sample	%Labeled Amount								
	Day 0	Day 3	Day 7	Day 14	Day 21	Day 30	Day 45	Day 60	Day 180
1	102.71	99.31	104.16	103.10	104.26	102.75	104.47	101.55	95.06
2	99.19	102.16	103.98	102.34	101.85	103.25	105.56	103.59	96.91
3	102.23	103.04	103.18	101.75	103.16	100.91	105.77	99.47	97.28
4	100.42	99.14	100.62	102.62	102.52	104.19	104.51	99.73	95.33
5	101.19	102.82	102.33	102.14	101.08	104.66	104.69	98.92	95.25
Mean *	101.15	101.30	102.85	102.39	102.57	103.15	105.00	100.65	95.97
SD	1.4111	1.92	1.44	0.51	1.22	1.46	0.62	1.92	1.09
	%Remaining								
Mean *	100.00	100.14	101.68	101.23	101.41	101.98	103.81	99.51	94.87
SD	1.39	1.90	1.43	0.50	1.21	1.45	0.61	1.89	1.03

* Significant differences between days (*p* < 0.05) were determined using a one-way ANOVA.

## Data Availability

Data are contained within the article.

## Abbreviations

The following abbreviations are used in this manuscript:

AOAC	Association of Official Analytical Collaboration
°C	Celsius degree
cm	Centimeter
CSOM	chronic suppurative otitis media
HCl	Hydrochloride
HPLC	High Performance Liquid Chromatography
HSP	Hansen Solubility Parameters
ICH	The International Council for Harmonisation of Technical Requirements for Pharmaceuticals for Human Use
Kg	Kilogram
L	Litter
LOD	Limit of Detection
LOQ	Limit of Quantitation
mL	Milliliter
Mol	Mole
MW	Molecular weight
NaOH	Sodium hydroxide
NF	National Formulary
pH	Potential of Hydrogen
pHp	precipitation pH
pKa	Acid dissociation constant
SD	Standard deviation
TSB	Tryptic soy broth
USP	United State Pharmacopeia
δ_d_	Dispersion force
δ_h_	Hydrogen bonding energy
δ_p_	Dipolar intermolecular force
µg	Micro gram
µM	Micro molar
%RSD	Percentage relative standard deviation
